# Setting Times of Early-Age Mortars Determined from Evolution Curves of Poisson’s Ratio

**DOI:** 10.3390/ma15030853

**Published:** 2022-01-23

**Authors:** Bate Bate, Xiao Chen, Chao Chen, Hongyan Ma, Jianfeng Zhu, Junnan Cao, Jiakai Chen, Kamal H. Khayat, Shuai Zhang

**Affiliations:** 1 Institute of Geotechnical Engineering, College of Civil Engineering and Architecture, MOE Key Laboratory of Soft Soils and Geoenvironmental Engineering, Zhejiang University, Hangzhou 310058, China; batebate@zju.edu.cn (B.B.); 21712211@zju.edu.cn (X.C.); chao_chen@zju.edu.cn (C.C.); 11912029@zju.edu.cn (J.C.); 2 Department of Civil, Architectural and Environmental Engineering, Missouri University of Science and Technology, Rolla, MO 65401, USA; mahon@mst.edu; 3 Pingan Bank, No. 5047 Shennan East Rd., Luohu District, Shenzhen 518010, China; jack.jianfeng.zhu@hotmail.com; 4 Department of Civil Engineering and Construction, Georgia Southern University, 1332 Southern Drive, Statesboro, GA 30458, USA; jcg83@mst.edu; 5 University Transportation Center and the Center for Infrastructure Engineering Studies, Department of Civil, Architectural and Environmental Engineering, Missouri University of Science and Technology, Rolla, MO 65409, USA; kkhayat@mst.edu

**Keywords:** mortar, setting time, Poisson’s ratio, P-wave velocity, dynamic elastic modulus

## Abstract

Setting times, as the early-age properties of cement-based materials, are important properties to ensure the quality and long-term performance of engineering structures. To determine the initial and final setting times of cementitious materials, the compressive wave velocity and shear wave velocity of six early-age mortar samples were monitored. Their time evolution curves of Young’s modulus, shear modulus, bulk modulus, and Poisson’s ratio were then calculated and analyzed. The signature times of the derivatives of the Poisson’s ratio evolution curves correlate well with the initial and final setting times, and the remarkably high coefficient of determination values relative to the data from this study are higher than those presented in the current literature. The proposed derivative method on the Poisson’s ratio evolution curve is as good as the derivative methods from vs. evolution curves used by prior studies for the estimation of both the initial and final setting times of the early-age properties of cement-based materials. The formation and subsequent disappearance of ettringite of low Poisson’s ratio were postulated to cause the initial dip in the Poisson’s ratio evolution curves.

## 1. Introduction

In cementitious materials, workability, durability, and mechanical properties are essential for quantifying the performance of structures [1,2,3,4,5,6,7]. Furthermore, to ensure the quality and long-term performance of facilities made from cement-based materials, determination of the early-age properties of such materials is important [8]. The initial and final setting times are early-age properties of cement-based materials, and thus they are important for construction projects using cementitious materials. The initial setting time (*t_i_*) refers to the time that elapses from the moment of the mixing of the cement with water to when the cement paste loses plasticity and becomes a semisolid material. The final setting time (*t_f_*) refers to the time that elapses from the moment the water is added to the cement to when the cement paste begins to gain strength. The current criteria used to measure the setting times are the penetration resistance test [9] for mortar or concrete and the Vicat needle test [10] for cement paste. However, both methods are destructive to the samples and cannot be used for actual structural components. Thus, alternative, non-destructive methods—which can be embedded in or attached to facilities, such as structures and pavement—are desirable.

In past decades, non-destructive, mechanical wave-based methods have been increasingly used to monitor early-age cement-based materials, and there have been some attempts to determine the setting times. These methods, as summarized in Table 1, include the ultrasonic pulse velocity method [6,7,8,9,10,11,12,13,14,15,16,17], the ultrasonic wave reflection method [18,19,20,21], the piezoceramic ring method [13,14,15,16,17,18,19,20,21,22,23,24], and in the FreshCon system developed by Reinhardt at the University of Stuttgart [24,25,26,27,28], the piezoceramic plate method (bender element) [29,30,31,32], the elasticity modulus measurement through ambient response method [33] and non-contacting electrical resistivity measurement technology [12,15].

As is shown in Table 1, time evolution curves of the compressive wave velocity (*V_p_*), shear wave velocity (*V_s_*), elastic modulus (*E*), and wave attenuation have often been obtained from the aforementioned methods. Correlations between the signature times obtained from the derivatives of the *V_p_* evolution curves and setting times are often only valid for materials with the same hydration processes and with the same gradation of solid particles [31], while the signature times obtained from the derivatives of the vs. evolution curves are better correlated with *t_i_* and *t_f_* [22,23,29,30], as was verified by the penetration resistance tests performed by many prior studies [18,19,20,21,22,23,24,25,26,27,28,29,30,31,32]. Moreover, the inflection time of the dynamic elastic modulus (*E_d_*) evolution curve was well correlated to *t_f_* with *R*^2^ ≥ 0.886, as was reported by Carette and Staquet after comparison with the results from the penetration resistance method [27]. In addition, Wang et al. [1] proposed an embedded guided waves method to monitor the setting process of mortar and concrete through continuous attenuation monitoring of the shear waves. It was found that the guided waves had high potential to determine the initial setting times of the cementitious materials [1]. Ghodousi et al. [34] developed a rough surface plate with the shear force and weight measurement method to determine the initial setting time of cement paste and concrete, and confirmed that the results from this method were comparable with Vicat tests.

To evaluate the early-age properties of cementitious materials, their elastic moduli (*E*), shear moduli (*G*), bulk moduli (*K*), and Poisson’s ratios (*ν*) are also needed and can be calculated from the *V_p_* and vs. evolution curves under the elastic material assumption, Equations (1–4) [27]:

(1) $$G=\rho V_{s}{}^{2}$$

(2) $$E_{d}=V_{p}{}^{2}\rho \frac{\left(1+\nu )(1-2\nu \right)}{1-\nu }$$

(3) $$K=\rho V_{p}{}^{2}-\frac{4}{3}G$$

(4) $$\nu =\frac{V_{p}{}^{2}-2V_{s}{}^{2}}{2V_{p}{}^{2}-2V_{s}{}^{2}}$$

It was noted that the values of *E*, *G*, *K*, and *ν* calculated from *V_p_* and vs. are essentially all high frequency dynamic properties, which could be related to their low frequency counterparts by the Kramers–Kronig relationship [35]. Accordingly, a subscript *d* should be added to these four symbols to denote their high-frequency nature, such as *E_d_*. However, because no low-frequency counterparts of these dynamic parameters are investigated in this work, the subscript *d* was omitted for *G*, *K*, and *ν* for the sake of simplicity. Although there has been preliminary success reported in the literature, the lack of an in-depth understanding of the wave transmission process while measuring the *V_p_* and vs. of cementitious materials at early ages remains the major obstacle. To obtain reliable high-frequency dynamic properties with wave methods, two factors should be considered for the selection of sample dimensions in a piezoceramic-based vs. measurement method, as elaborated by Bate et al. [31] and Kang et al. [36]: (1) There is an inherent system lag in shear wave velocity measurement. Therefore, a long travel distance is required to ensure sufficient travel time to offset the time lag; (2) the wave travel distance should match the excitation energy, as the amplitude of the received wave signals attenuates quickly over travel distance. In addition, the excitation frequency should be in close proximity to the resonance frequency of the specimen to obtain clear received signals and a high signal-to-noise ratio [36]. Neglect of these requirements results in vs. signals that are limited to short time spans, which are insufficient to determine the setting times and reveal the plateau values of the vs. evolution curves.

Building upon prior studies by the same authors on vs. measurements of six mortar samples over the complete curing stages, the objectives of this study were to: (1) measure the *V_p_* evolution curves of six mortars; (2) calculate the time evolution curves of *E_d_*, *G*, *K*, and *ν* from the measured *V_p_* and vs. data; (3) calculate the derivatives of these evolution curves, identify the characteristic times, and correlate them with the measured setting times; and (4) compare these correlations with existing ones and provide significant findings for engineering practice.

## 2. Materials and Experimental Methods

### 2.1. Materials

The materials used in this study were the same as those used in our previous study [30], and a brief description is provided. A Type I Portland cement complying with ASTM C 150 [37] was adopted for all the tests. It contained 65% CaO, 21.1% SiO_2_, 6.2% Al_2_O_3_, 2.9% Fe_2_O_3_, and 2.0% SO_3_. The fine aggregate used in this study was a Missouri River sand whose D_50_ was 0.7 mm, and the coefficient of uniformity (D_60_/D_10_) was 2.74. In order to be comparable with prior studies, six mortar mixtures with *w*/*c* ranging from 0.37 to 0.5 were prepared in compliance with ASTM standards C494 [38] and C305 [39] under room temperature (around 22 °C) and approximately 40% humidity (Table 2). To expand the range of setting times, a non-chloride accelerating admixture and hydration retarder were used to shorten and prolong the setting times, respectively. More details of the materials used in this study can be found in our previous publication [30].

### 2.2. Ultrasonic Pulse Velocity Test

**Figure 1:**
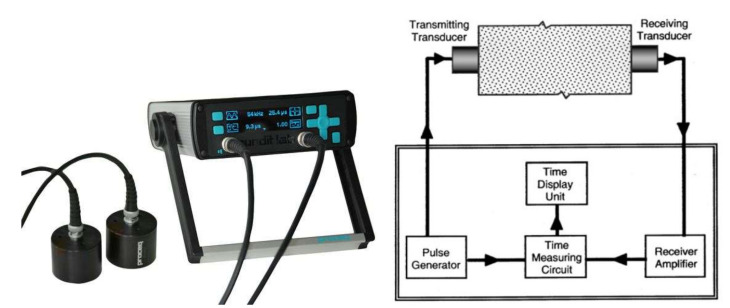
Ultrasonic pulse velocity device and setup.

The P-wave velocities of the cement-based materials at early ages were measured using an ultrasonic pulse velocity device (Figure 1) with a transport time resolution of 0.1 μs, which is in compliance with ASTM C597 [40]. Two transducers (standard 54 kHz transducers, Proceq Int., Aliquippa, PA) were placed into two pre-drilled holes (0.051 m in diameter, QUIKRETE, Atlanta, GA) on the wall of a cylindrical mortar container (0.203 m in diameter, QUIKRETE, Atlanta, GA). Vaseline was applied to the transducer faces and the specimen surfaces to ensure good contact. Transducers were firmly pressed against the surfaces of the mortar sample until a stable transmission time was reached. The time-elapsed counting began from the moment that the water was added to the cement. Readings of the transmission time were obtained every 1/2 to 6 h during the first 24 h, and subsequently every 2 to 10.5 h. The P-wave velocity was determined by dividing the travel distance by the recorded transmission time.

## 3. Results

### 3.1. V_p_ Evolution Curves

**Figure 2:**
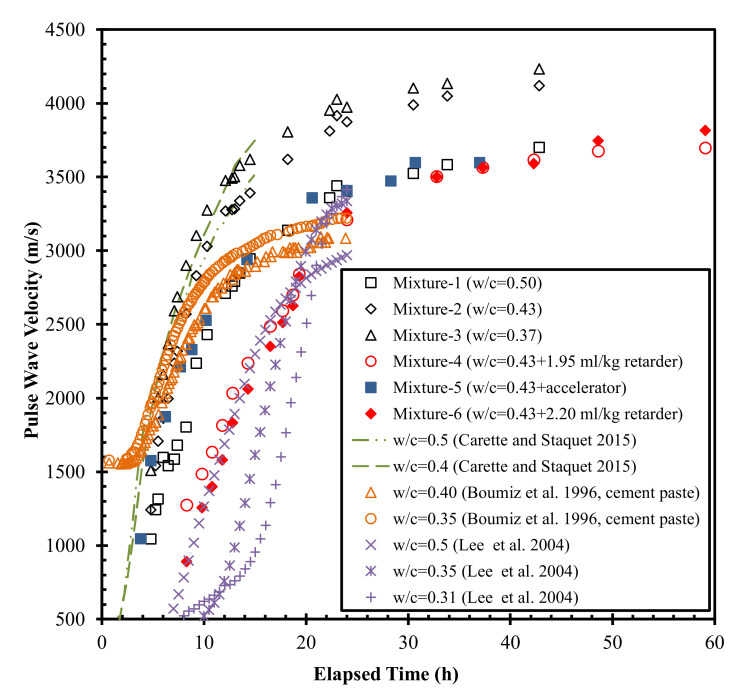
The compressive wave velocity *Vp* evolution curves.

The *V_p_* evolution curves collected in this study and from the literature are plotted in Figure 2. This figure shows that *V_p_* increased monotonically until it reached a plateau value at ages greater than around 30 h. The plateau value increased with the decrease in water-to-cement ratio (*w*/*c*). This correlates with the findings of previous studies on mortars and cement pastes [26,32]. The plateau value of *V_p_* ranged from 3153 to 4232 m/s for the six mortars, which is comparable to those reported in [41] (3000 to 3500 m/s) for typical mortars with similar *w*/*c* of 0.3 to 0.6 (Figure 2). In particular, the *V_p_* plateau value (approximately 3600 m/s) of Mixture-1 with a *w*/*c* of 0.50 was comparable (3520 m/s) to that of a previously studied mortar at the same *w*/*c* ratio reported by Carette and Staquet [27]. Figure 2 also indicates that the use of a set accelerator initially increases the *V_p_* of the mortar sample (the first 6–7 h) compared with the reference mortar, then decreases it until it approaches an asymptotic value. The incorporation of the set retarder decreased *V_p_* throughout the curing process. The differences of *V_p_* between Lee et al. [26] and this study in Figure 2 might be due to: (1) Lee et al. [26] used 20% weight of low-calcium FA produced in coal-fueled electric power station as the cementitious materials, and (2) different experimental environments (e.g., temperature, humidity, etc.). Even though the curve trends of both studies are different, the plateau values of *V*_p_ after around 20 h are comparable with each other.

### 3.2. Fitting Methods and Parameters

As the *V_p_* and vs. values were not measured simultaneously, fitting of their evolution curves was necessary to calculate *E_d_*, *G*, *K*, and *ν*. The measured *V_p_* values of early-age mortars obtained by Lee et al. [26], Carette and Staquet [27], and this study and the measured vs. values reported by Carette and Staquet [27] were fitted using a lognormal cumulative Equation (5):

(5) $$y=b\left(0.5+0.5\times \mathrm{erf}\frac{lnx-\mu }{\sqrt{2}\sigma }\right)+c,$$

where *μ*, *σ*, and *c* are fitting parameters and *b* is the maximum number. The vs. values of the six mortars in this study were fitted previously [30] using a modified Fredlund and Xing [42] equation, which features a reverse S-shape with three independent parameters to dictate the onset of initial bending (*a*), the slope of the major increment (*n*), and the plateau slope (*m*), respectively Equation (6):

(6) $$y=\theta_{s}-\theta_{s}{\left[\frac{1}{\ln(e+{\left(\psi /a\right)}^{n}}\right]}^{m},$$

**Figure 3:**
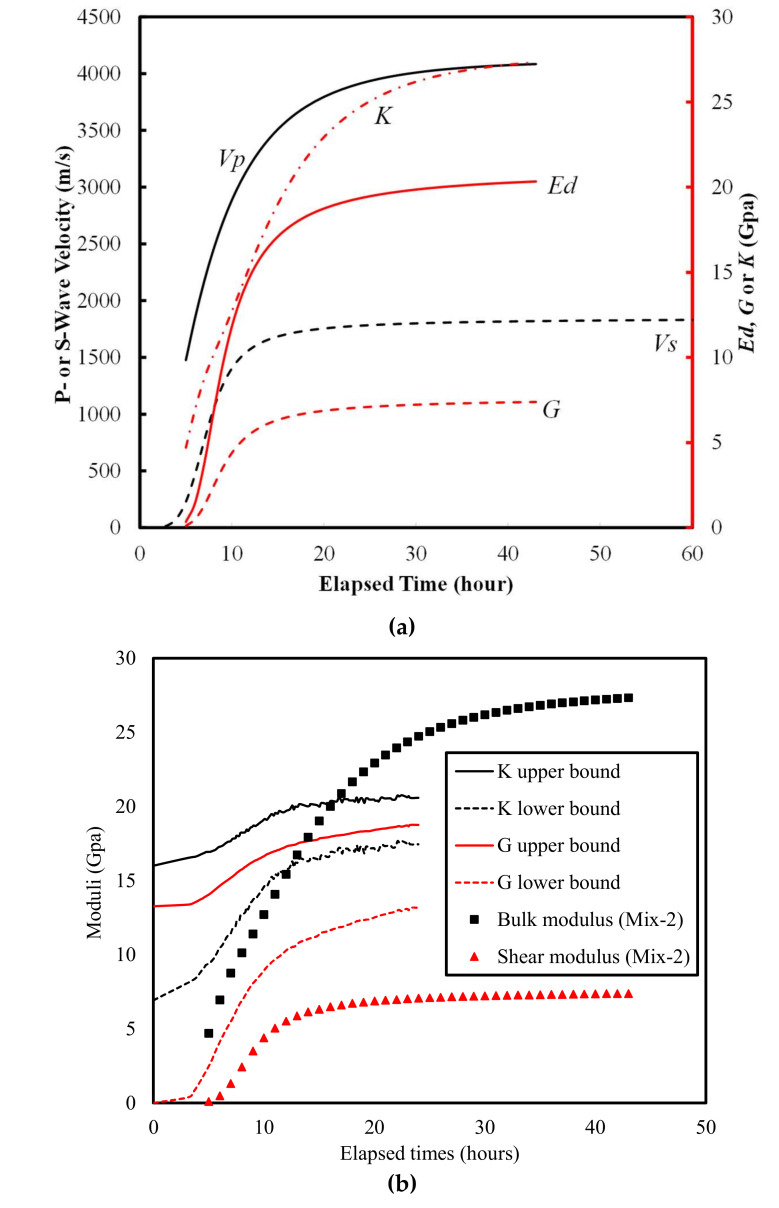
(**a**) Representative evolution curves of *V*_s_, *V*_p_ (black lines), (**b**) *E*_d_, *G*, and *K* (red lines) for Mixture-2 (w/c = 0.43).

Finally, the *E_d_* evolution curves obtained using the fit *V_p_* and V_s_. values were fitted using a lognormal cumulative equation. The fitting results are illustrated in Figure 3 and summarized in Table 3.

### 3.3. E_d_, G, K, and ν Evolution Curves

**Figure 4:**
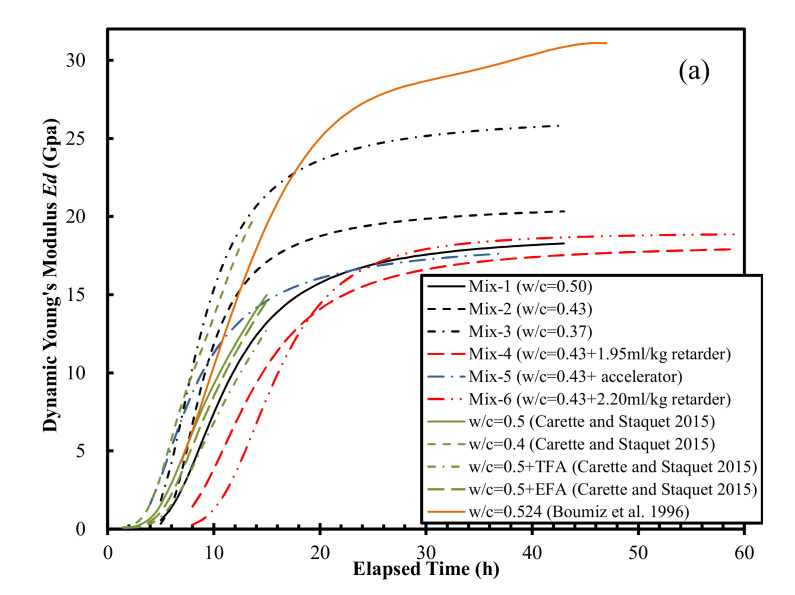

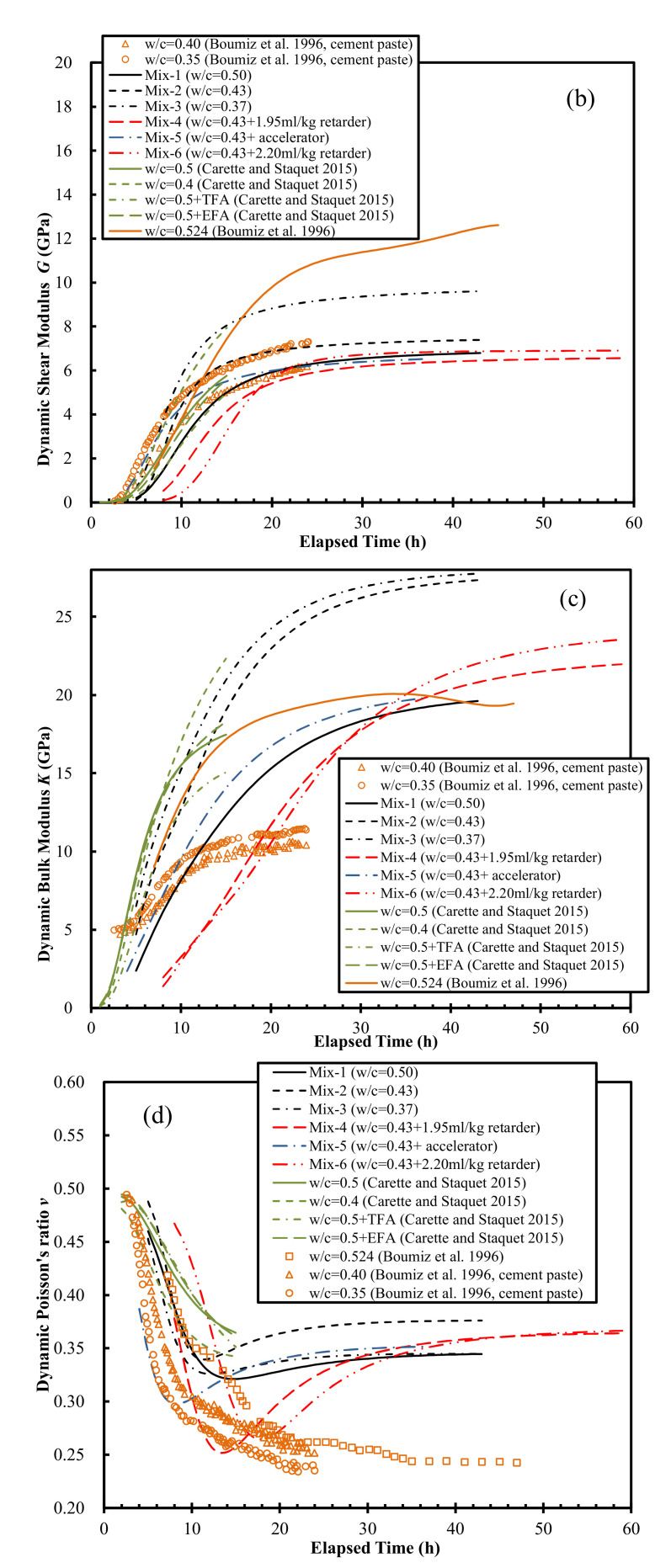
Evolution curves of (**a**) Ed, (**b**) G, (**c**) K, and (**d**) ν. Note: G = 5.9 GPa for hardened mortar [45]; G = 10.6 Gpa for hardened mortar with a w/c of 0.50 [44]; K = 47 ± 4 Gpa for hardened concrete [46].

The calculated evolution curves of *E_d_, G, K,* and *ν* are shown in Figure 4. The following observations can be made from this. The evolution curves of *E_d_*, *G*, and *K* were all S-shaped, except for those reported by Carette and Staquet [27], where the asymptotic values were not obtained. Lower *w*/*c* corresponded to higher values of *E_d_*, *G*, and *K*, which is consistent with previous results [27,32]. The Poisson’s ratio first decreased from approximately 0.50 (that of a liquid) to a minimum value ranging from 0.25 to 0.33, after which it increased to a steady-state value ranging from 0.34 to 0.38 (Figure 4d). These steady-state values are larger than previously reported values for mortar samples, namely 0.21 [43], 0.24 [32] and 0.29 [44], but are close to the range (0.35 to 0.37) reported by Carette and Staquet [27]. The “local valley” in the Poisson’s ratio evolution curves in this study have not been reported previously. The possible mechanisms are discussed in the Discussion section.

In addition, there was no consistent relationship between Poisson’s ratio and the *w*/*c* or curing age, which correlates with the observations of Mehta and Monteiro [47].

## 4. Discussion

### 4.1. Setting Times Determined from Derivatives of Poisson’s Ratio Evolution Curves

**Figure 5:**
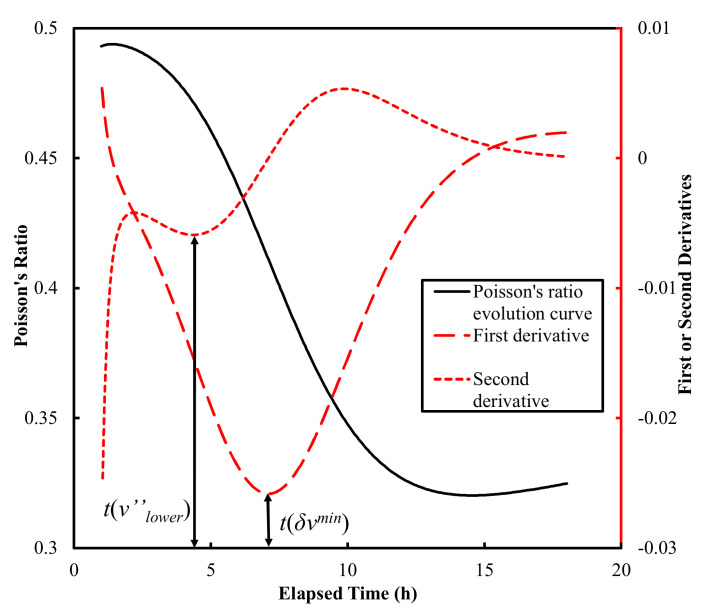
Process of estimating setting times by Poisson’s ratio derivative method.

First and second derivatives of the Poisson’s ratio evolution curves reported by Carette and Staquet [27] and those obtained in this study were calculated using a differential method with time steps of 3 min (Figure 5).

**Figure 6:**
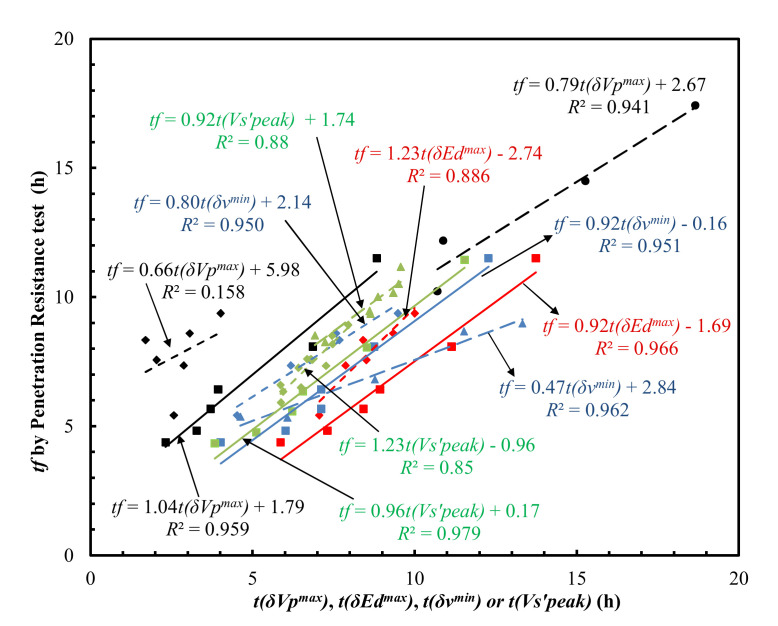
Comparison of t(δVpmax), t(δEdmax), t(δνmin) or t(Vs’peak) with *t_f_* determined by the penetration resistance test. Note: Square, diamond, circular, triangle, ‘×’ and ‘+’ symbols represent the experimental data from this study, Carette and Staquet [27], Lee et al. [26], Carette and Staquet [28], Naji et al. [22] and Naji et al. [23], respectively.

The inflection point *t*(*δν^min^*) was subsequently used to correlate the final setting times, which yielded *R*^2^ values of 0.951, 0.962, and 0.950 for the data in the two reports by Carette and Staquet [27,28] and in this study, respectively (Figure 6).

**Figure 7:**
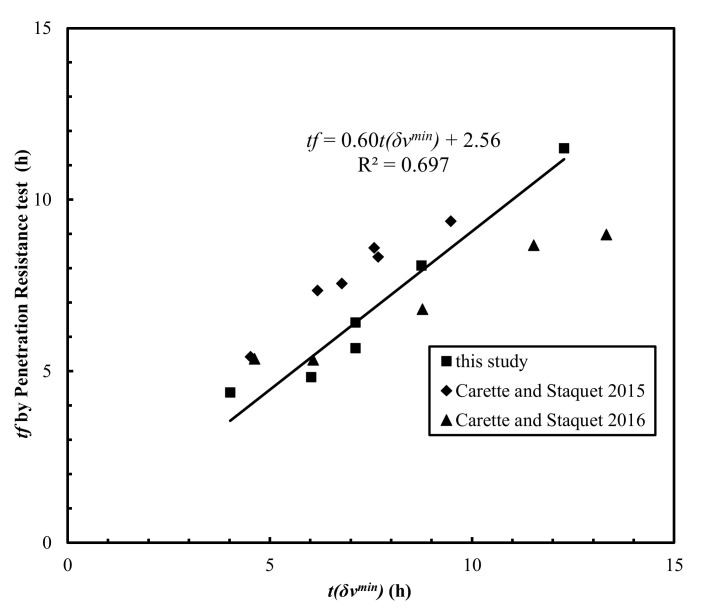
Comparison of *t*(*δν^min^*) and *t_f_* obtained by the penetration resistance test.

However, linearly fitting all the data points of *t*(*δν^min^*) and experimental values of *t_f_* from these studies yielded an *R*^2^ value of 0.697 (Figure 7).

**Figure 8:**
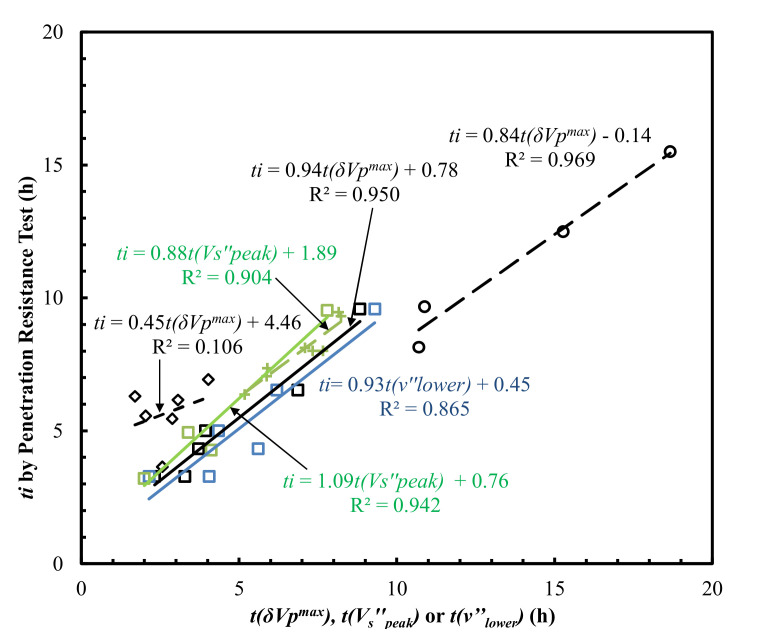
Comparison of *t*(*δV_p_^max^*), *t*(*ν’’_lower_*) or *t*(*V_s_^’’^_peak_*) with *t_i_*, determined by the penetration resistance method. Note: Square, diamond, circular, and ‘+’ symbols represent the experimental data from this study, Carette and Staquet [27], Lee et al. [26], and Naji et al. [23], respectively.

The lower *R*^2^ value could be due to the different material properties, testing setups, or sample dimensions used in those studies. For example, the possible inaccuracy caused by uncalibrated system lag time due to the short travel distances used in Carette and Staquet [27,28], as discussed by Bate et al. [31], was accounted for by subtracting the calibrated system lag time from the measured travel time in this study. More measurements of the Poisson’s ratio evolution curves were warranted for the assessment of the reliability of the proposed Poisson’s ratio derivative method. The lower concave point *t*(*ν’’_lower_*) proceeding the inflection point was used to correlate the initial setting times (Figure 8), which yielded *R*^2^ = 0.865 for the data obtained in this study. It was noted that the *V_p_* of Mix-3 and Mix-5 after the first 4 h is difficult to measure.

Alternatively, *V_p_* was extrapolated from its fitted time-evolution curve. Consequently, the initial setting times estimated by the second derivative method of Poisson’s ratio, in which the Poisson’s ratio was calculated by *V_p_*, could be less accurate than those estimated from actual experimental data.

These preliminary results suggest that the Poisson’s ratio derivative method can predict setting times, especially final setting times, reasonably well. But this method is suggested for use under conditions of the same testing setup, testing environment, cement and aggregate.

**Figure 9:**
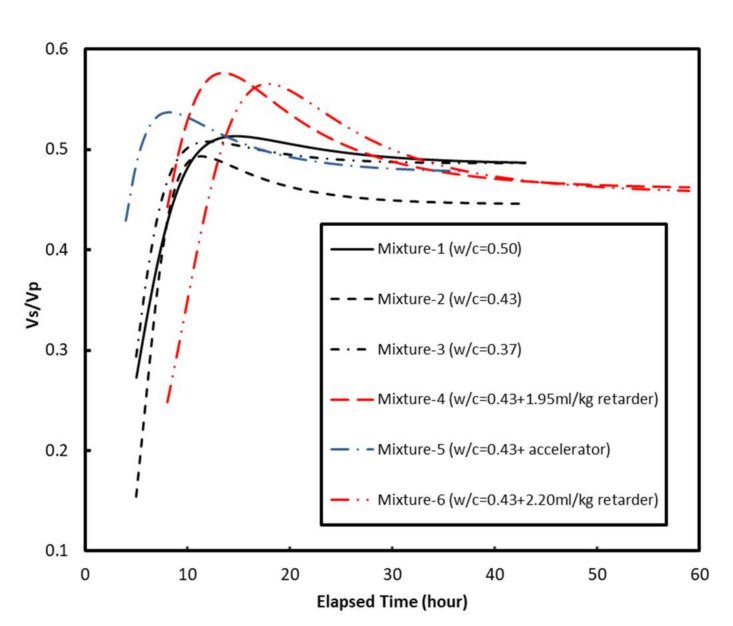
Evolution curves of *V_s_*/*V_p_* for Mixtures 1 to 6.

It is widely accepted that the moduli of elasticity of cement-based materials increase monotonically with time, as shown in Figure 4. However, in this study the values of Poisson’s ratio exhibited non-monotonic behavior—first decreasing and subsequently increasing, separated by the “local valley”, as shown in Figure 4d. This is because the ratio of *V*_s_ to *V_p_* of six tested mortars firstly increased, then decreased (Figure 9), and the Poisson’s ratio is negative correlated with it (Equation (4)), so that the “local valley” appeared in Poisson’s ratio evolution curves. However, the above is an explanation of the results obtained from this experiment; a mechanism level explanation for the “local valley” phenomenon is conducted below.

Cement paste starts as a suspension, and early hydration before the final setting is a transition process from liquid/semisolid to semisolid/solid. This transition from softer to harder matter could lead to a decrease in Poisson’s ratio. On the other hand, a relaxation is caused by the time-dependent dissolution of cement grains, which decreases the Poisson’s ratio of the cement paste [48]. Meanwhile, early hydration is dominated by ettringite formation, which consumes C_3_A and gypsum. Because the Poisson’s ratios of both solid reactants (0.3 and 0.34) are higher than that of ettringite (0.25), as reported by former studies [46,47,49,50,51,52], this reaction should also be partially responsible for the decrease in Poisson’s ratio. During the early hydration before the final setting, solid phases are unlikely to be well-connected and form a percolated rigid network. Therefore, cement paste can be considered a gradually thicker solid suspension during this period. During a later hydration stage after the final setting, a well-connected solid skeleton is formed. Thereafter, ettringite reacts with excessive C_3_A to form monosulfoaluminate, whose Poisson’s ratio (0.324) [49] is higher than ettringite, and C-S-H and CH continuously form owing to the hydration of calcium silicate phases, filling capillary pores in the solid skeletal structure and densifying the interfacial zone between the bulk paste and aggregate. The phase transition and pore filling/densifying effect result in the increase in Poisson’s ratio. The inflection point *t*(*δν^min^*) in Figure 5 indicates the start of a transition from the early hydration stage to a later hydration stage, as discussed earlier, and thus it can be correlated with the final setting time. The correlation between the *t*(*ν’’_lower_*) and initial setting time is established empirically. Further study is necessary to identify the physical correlations between the initial setting and the characteristic points on the experimentally obtained curves.

### 4.2. Comparison with Derivative Methods on V_s_, E_d_, V_p_, K and G Evolution Curves

**Figure 10:**
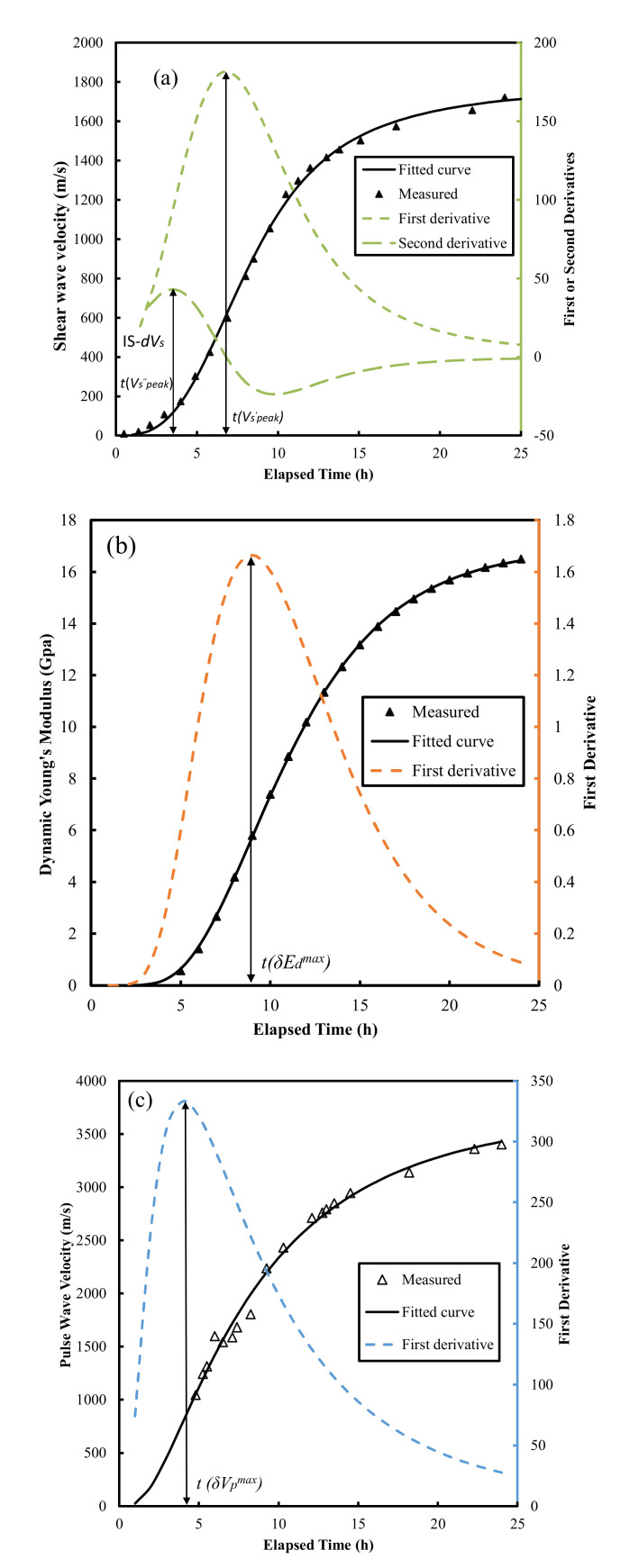
Procedures for estimating (**a**) the initial and final setting times by the vs. derivative method, (**b**) the final setting times by *E_d_* derivative method, and (**c**) the initial and final setting times by *V_p_* derivative method. Note: IS-dVs, the time at the beginning moment of S-wave, was proposed to correlate the initial setting times by Naji et al. [22].

The derivative methods on *V*_s_ evolution curves (Figure 10a) yield high correlations with final setting times (Figure 6) with an *R*^2^ of 0.858–0.979 (Naji et al. [22,23], Zhu et al. [30]), and initial setting times (Figure 8) with an *R*^2^ of 0.904–0.950 (Naji et al. [23], Zhu et al. [30]). The results outlined above suggest that, similar to the Poisson’s ratio method proposed in this study, the vs. derivative method predicts both the initial and final setting times with a high coefficient of determination (*R*^2^). It should be noted that the shear modulus (*G*) evolution curves (Figure 3b) could also be correlated with the setting times, given the intrinsic relationship between *G* and shear wave velocity (*V_s_*): *G* = bulk density × $V_{s}^{2}$.

The times of the inflection points *t*(*δE_d_^max^*) of the *E_d_* evolution curves (Figure 10b) also correlated well with the final setting times (Figure 6), which yielded *R*^2^ values of 0.886 and 0.966 for the data reported by Carette and Staquet [27] and from this study, respectively. However, the *E_d_* derivative method has only been used for predicting final setting times [27,28].

The time, *t*(*δV_p_^max^*), corresponding to the peak value of first derivatives of the *V_p_* evolution curves (Figure 10c) in Carette and Staquet [27], Lee et al. [26] and this study, correlates with both the initial and final setting times (Figure 6 and Figure 8) with wide *R*^2^ ranges, from 0.106 to 0.969 and from 0.158 to 0.959 for the initial and final setting times, respectively. This suggests that the *V_p_* derivative method was very material-specific [44] and was inconsistent. The setting times of the six mortars determined by the five different methods outlined above (Poisson’s ratio, *V_s_*, *E_d_*, *V_p_* derivative methods and penetration resistance method) are summarized in Table 2.

The inflection point *t*(*δK^max^*) of the *K* evolution curves (data not shown here) correlated well with the setting times used in this study (*R*^2^ ranged from 0.961 to 0.971) but poorly for the data reported by Carette and Staquet [27] (*R*^2^ ranged from 0.072 to 0.135). These inconsistent results suggest that the *K* derivative method is also material-specific.

In summary, compared with the derivative on the *E_d_* evolution curve, which is only correlated with the final setting time of cementitious materials, the derivatives on *V*_s_, *G*, and Poisson’s ratio evolution curves predicted both initial and final setting times very well, with a relatively high coefficient of determination (*R*^2^ ≥ 0.858). Even though *V*_p_ and *K* could be used to predict the setting times, they are very material-specific, yielding inconsistent results.

## 5. Conclusions

A novel approach for determining the initial and final setting times of early-age cementitious materials was proposed by the derivatives of the time evolution curve of Poisson’s ratio. The Poisson’s ratio was determined using the compressive wave velocity evolution curves of six early-age mortar specimens measured in this study, together with a prior study of the shear wave velocity evolution curves of the same materials under the same conditions. The evolution curves of the dynamic elastic modulus (*E_d_*), dynamic shear modulus (*G*), dynamic bulk modulus (*K*), and dynamic Poisson’s ratio (*ν*) were obtained and analyzed. The characteristic times on these curves and their first/second derivative curves were correlated with the setting times. The major findings are:

(1) The signature times of the derivatives of the Poisson’s ratio evolution curves were found to correlate well with the initial and final setting times, with *R*^2^ values of 0.865 and 0.950–0.962, respectively, based on the data reported by Carrette and Staquet (2015, 2016) and by this study. The derivative method on the Poisson’s ratio evolution curve is as good as the derivative methods from shear wave velocity evolution curves used by Naji et al. (2017, 2018) and Zhu et al. (2018). The two methods are recommended for estimation of both the initial and final setting times of the early-age properties of cement-based materials.
(2) The signature times of the inflection points of the *E_d_* evolution curves correlated well with the final setting times measured by standard penetration tests, with *R*^2^ values of 0.966 (data in this study) and 0.886 (data in Carette and Staquet, 2015), respectively. Therefore, the derivative method on the *E_d_* evolution curve is validated by this study, and is recommended for the estimation of the final setting time of the early-age properties of cement-based materials.
(3) During the early hydration stage, the C_3_A and gypsum were consumed and ettringite of comparatively lower Poisson’s ratio (0.25) was formed. This is postulated to cause the initial dip in the Poisson’s ratio evolution curves of all six mortar specimens tested in this study. After the final setting, excessive C_3_A reacted with ettringite, and monosulfoaluminate of higher Poisson’s ratio (than ettringite) was generated. Monosulfoaluminate generation, together with the pore filling/densifying effect caused by the hydration of calcium silicate phases, was postulated to cause further increment in the Poisson’s ratio.
(4) The preliminary results of this study suggested that the Poisson’s ratio derivative method could predict setting times of cementitious materials, especially the final setting times, reasonably well. But this method is suggested for use under the specific conditions of this study, i.e., the same testing setup, the same testing environment, the same cement and aggregate. Further measurements of other cementitious materials, including a variety of water-cement ratios and mortar with different binders, are necessary for the assessment of the reliability of the proposed method in the future.

## Figures and Tables

**Table 1:** Summary of nondestructive testing methods for initial and final setting times.

Method	Time Evolution of	1st Derivative	2nd Derivative	*t_i_*	*t_f_*	References	Note *
Ultrasonic pulse velocity	*V_p_*	Y	-	Y	-	[11,12,13,14,15,16,17]	Degree of agreement with Vicat needle method varies.
Ultrasonic wave reflection	*V_s_*	Y	-	Y	Y	[18,19,20,21]	*R*^2^ = 0.82
Piezoceramic ring	*V_s_*	Y	-	Y	Y	[22,23]	*R*^2^ = 0.89
FreshCon system	*V_s_* & *V_p_*	Y	-	Y	Y	[24,25,26,27,28]	*R*^2^ = 0.106–0.969 (*t_i_*), *R*^2^ = 0.158–0.959 (*t_f_*)
Piezoceramic plate (bender element)	*V_s_*	Y	Y	Y	Y	[29,30,31,32]	*R*^2^ = 0.742–0.950 (*t_i_*), *R*^2^ = 0.861–0.979 (*t_f_*)
Elasticity modulus (*E_d_*)	*E_d_*	Y	-	-	Y	[27,28,33]	*R*^2^ = 0.886–0.966 (*t_f_*)
Electrical resistivity	electrical resistivity	Y	-	Y	Y	[14,15]	*R*^2^ = 0.895 (*t_i_*), 0.989 (*t_f_*)
Poisson’s ratio (*ν*)	*ν*	Y	Y	Y	Y	This study	

*: all *R*^2^ values are being compared with penetration resistance method; Y: method used, or value predicted.

**Table 2:** Details of mixture design and setting times (*h*) calculated by the *V*_p_, *V*_s_, *E*_d,_ and *ν* derivative method and penetration resistance test of the six mortar mixtures.

Mix Design	Mixture-1	Mixture-2	Mixture-3	Mixture-4	Mixture-5	Mixture-6
*w*/*c*	0.5	0.43	0.37	0.43	0.43	0.43
Cement (kg/m^3^)	673	713	751	713	713	713
Sand (kg/m^3^)	1137	1203	1267	1203	1203	1203
Water (kg/m^3^)	337	313	277	313	313	313
Unit weight (kg/m^3^)	2177.5	2273.5	2309.2	2253.0	2222.4	2253.0
Retarder (mL/100 kg of cement)	--	--	--	195	--	220
Accelerator (mL/100 kg of cement)	--	--	--	--	1500	--
*t*(*δV_p_^max^*) (h)	3.94	3.71	3.28	6.86	2.32	8.83
*t*(*ν’’_lower_*) (h)	4.35	5.6	4.05	6.2	2.15	9.30
*t*(*V_s_^’’^_peak_*) (h)*	4.59	5.32	3.03	6.78	3.13	9.17
*t_i_* (h)	5	4.33	3.28	6.53	3.28	9.58
*t*(*δV_p_^max^*) (h)	3.94	3.71	3.28	6.86	2.32	8.83
*t*(*δE_d_^max^*) (h)	8.93	8.42	7.31	11.14	5.87	13.74
*t*(*δν^min^*) (h)	7.12	7.12	6.02	8.75	4.02	12.28
*t*(*V_s_^’^_peak_*) (h)*	6.50	6.20	5.10	8.50	3.80	11.50
*t_f_* (h)	6.42	5.67	4.83	8.08	4.38	11.50

*: from Zhu et al. [30].

**Table 3:** Fitting parameters of lognormal and Modified Fredlund and Xing equations for experimental data of mortars in this study.

Type of Evolution Curves	Fitting Equation	Parameters	Mixture-1	Mixture-2	Mixture-3	Mixture-4	Mixture-5	Mixture-6
*V_p_* evolution curves	Lognormal	*Μ*	2.04	1.88	1.75	2.49	1.85	2.59
		*Σ*	0.82	0.76	0.75	0.75	1.00	0.64
		*v_p,max_*	3738.4	4071.7	4137.5	3806.8	3762.8	3809.5
		*C*	0.00	0.00	0.00	0.00	0.00	0.00
		*R* ^2^	0.992	0.996	0.994	0.997	0.996	0.997
*E_d_* evolution curves	Lognormal	*μ*	2.38	2.25	2.17	2.55	2.07	2.70
		*σ*	0.43	0.35	0.42	0.37	0.55	0.29
		*E_d,max_*	16.99	19.27	24.18	15.39	16.89	16.68
		*c*	0.00	0.00	0.00	0.00	0.00	0.00
		*R* ^2^	0.999	0.999	0.999	0.999	0.999	0.999
*V_s_* evolution curves *	Modified Fredlund and Xing	*a*	8.07	6.70	6.19	9.78	4.82	14.35
		*n*	3.01	5.20	3.46	3.68	2.68	4.05
		*m*	2.26	1.66	1.92	2.16	1.82	3.71
		*R* ^2^	0.999	0.995	0.999	0.997	0.998	0.999

*: from Zhu et al. [30].
